# Incremental Health Care Costs Associated With Food Insecurity and Chronic Conditions Among Older Adults

**DOI:** 10.5888/pcd15.180058

**Published:** 2018-08-30

**Authors:** Sandra P. Garcia, Anne Haddix, Kevin Barnett

**Affiliations:** 1 Public Health Institute, Oakland, California; 2 Inequality and Policy Research Center, Claremont Graduate University, Claremont, California; 3 CDC Foundation, Atlanta, Georgia; 4 Minga Analytics, LLC, Savannah, Georgia

## Abstract

**Introduction:**

The prevalence of food insecurity and chronic health conditions among older adults is a public health concern. However, little is known about associated health care costs. We estimated the incremental health care costs of food insecurity and selected chronic health conditions among older adults, defined as adults aged 50 or older.

**Methods:**

We analyzed 4 years of data (2011–2014) from the National Health Interview Survey and 3 years of data (2013–2015) from the Medical Expenditure Panel Survey; we used 2-part models to estimate the incremental health care costs associated with food insecurity and 9 chronic conditions (hypertension, coronary heart disease, stroke, emphysema, asthma, cancer, chronic bronchitis, arthritis, and diabetes) among older adults.

**Results:**

Approximately 14% of older adult respondents (n = 2,150) reported being food insecure. The 3 most common chronic conditions were the same for both food-insecure and food-secure older adults: hypertension, arthritis, and diabetes. The adjusted annual incremental health care costs resulting from food insecurity among older adults were higher in the presence of hypertension, stroke, and arthritis (*P* ≤ .05) and in the presence of diabetes (*P* ≤ .10). These findings were also true for the incremental health care costs resulting from food insecurity in the absence of these specific chronic conditions.

**Conclusion:**

Our findings show that food insecurity interacts with chronic conditions. We observed higher health care costs in the presence of this interaction for those who were food insecure and had poor health than for those who were food secure.

## Introduction

Food insecurity — the inability to access sufficient and nutritious food for an active, healthy life — has been identified as a critical social factor that carries negative nutritional, physical, and psychological health implications for adults (1). More than 29 million adults experienced food insecurity in 2015 (2). Over the past 2 decades, the prevalence of food insecurity in US households remained above 10%. Research suggests that from 2005 through 2025 the food insecurity rate will increase by 75% for adults aged 60 or older (3).

There is increasing evidence that food insecurity is associated with chronic health conditions, including diabetes, hypertension, asthma, arthritis, chronic bronchitis, and emphysema (4–8). This link may be particularly strong among older adults, defined as adults aged 50 or older, who are at higher risk of chronic conditions than younger adults (9). According to a 2014 report, compared with younger adults, food-insecure adults aged 60 or older were 53% more likely more likely to report a heart attack, 40% more likely to report congestive heart failure, 22% more likely to report coronary heart disease, and 52% more likely to develop asthma (10).

Although previous studies estimated the health care costs for food-insecure adults (11–15), little research exists examining the health care costs associated with food insecurity and chronic health conditions among older adults. To address this gap, we estimated the incremental health care costs associated with food insecurity and the existence of selected chronic health conditions among older adults.

## Methods

### Study data

Our analysis used pooled annual data from the 2013–2015 Medical Expenditure Panel Survey (MEPS) and the 2011–2014 National Health Interview Survey (NHIS) — 2 nationally representative surveys of the US civilian noninstitutionalized population. We linked data from the NHIS Sample Adult Core, which contains data on chronic conditions, and the NHIS Family Core, which contains data on food insecurity, whether or not an adult lives alone, and USDA Supplemental Nutrition Assistance Program (SNAP) participation, to MEPS records by using the MEPS/NHIS linkage file. The MEPS/NHIS linkage file merges each calendar MEPS year with the prior 2 years of NHIS data (16). MEPS data include a person’s annual total health care costs. MEPS also collects information on sociodemographic characteristics, including age, sex, race/ethnicity, region, family income as a percentage of the Federal Poverty Level, and health insurance status. All analyses incorporated MEPS sampling weights and both variance weights (primary sample unit and strata). Our analysis used complete data cases, removing from the analytic sample all observations with missing data or “unknown” answer data, for a final analytic sample of 14,879 observations.

### Measures

**Health care costs.** The 2013–2015 MEPS data provide records on the annual total costs for all health care services from all payment sources incurred per person. Health care costs were adjusted to 2015 US dollars by using the Agency for Healthcare Research and Quality’s Personal Health Care Expenditure Price Index (17).

**Food insecurity.** The 2011–2014 NHIS Family Core contains a set of 10 questions that assess adult food security in the past 30 days (18). For this analysis, we used the available FSSTAT variable, which was constructed based on the 10 food security questions. FSSTAT represents a 30-day food security scale. For the 2013 and 2014 NHIS, the FSSTAT variable was not available; thus, it was constructed following instructions provided in the 2011 NHIS Survey Description file (18). This variable offers 3 levels of food security: 1) food secure, 2) low food security, and 3) very low food security. We used the FSSTAT variable to construct a new 2-category variable: 1) food insecure and 2) food secure (19). This new variable combined the low and very low food security statuses into a single food-insecure status, whereas the food-secure status was left as it was in the FSSTAT variable. In quarters 3 and 4 of the 2013 NHIS, the wording in 3 of the 10 food security questions varied slightly. The original versions of these 3 questions asked about food security status in reference to the respondent only, whereas the modified version asked about the respondent and other adults in the household. To include these participants in the analytic sample, we added a dummy variable to control for subtle differences in the wording of the questions.

**Chronic conditions.** The 2011–2014 NHIS Sample Adult Core contains data on several self-reported chronic conditions. Adult respondents were asked if they had ever been told that they had a specific chronic condition. For chronic bronchitis, adult respondents were asked if they had the condition during the past 12 months. We consulted with experts in food insecurity and chronic conditions to select 9 chronic health conditions for our study. All these conditions were previously identified in the literature as associated with food insecurity or high health care use and costs (13,20–22). The 9 chronic health conditions were hypertension, coronary heart disease, stroke, emphysema, asthma, cancer, chronic bronchitis, arthritis, and diabetes.

**Sociodemographic and economic variables.** Age was categorized in 2 groups: 1) adults aged 50 to 64, and 2) adults aged 65 or older; adults over 85 were coded as age 85 (23). This was to account for possible differences in health, health insurance coverage, health care use, and food security status in adults aged 50 to 64 and those aged 65 or older. Respondents were categorized as male and female. Race/ethnicity was categorized into 4 groups: 1) non-Hispanic white, 2) non-Hispanic black, 3) Hispanic, and 4) non-Hispanic other. Health insurance coverage was divided into 3 groups: 1) private, 2) public, and 3) uninsured. Regional location consisted of 4 census regions: 1) Northeast, 2) Midwest, 3) South, and 4) West. Household size was categorized into 2 living arrangements: living alone or living with others. Poverty level was categorized as: 1) below 125% of the household size–specific Federal Poverty Level, 2) from 125% to 199% of the Federal Poverty Level, and 3) at least 200% of the Federal Poverty Level. Participation in SNAP, the major federal program for alleviating food insecurity, was indicated as whether or not the adult respondent had received SNAP benefits at any time during the last calendar year. Because we pooled data from different surveys across several years, we included year-fixed effects to hold constant determinants of health care costs that may vary across survey samples over time.

### Statistical analysis

We used an econometric approach to assess the incremental annual health care costs associated with food insecurity among adults aged 50 years or older with a chronic condition by using a 2-part model (24). This 2-part model was used to account for the large fraction of observations with zero total health care cost values and the right-skewed costs distribution resulting from observations with extremely high values (25). The first part of the 2-part model used the complete sample to estimate the probability of incurring a positive health care cost value. The second part estimated health care costs for people who manifested positive health care cost values. We used a logistic regression for the first part and a generalized linear model with a log link for the second part. We used a Poisson distribution in the second part. We used a modified Park test to identify the appropriate distribution (26). Predictions from each part of the model were subsequently multiplied by each other to calculate the incremental total health care costs.

The estimates of health care costs for all combinations of food insecurity status and having or not having a specific chronic condition were predicted separately by setting the food insecurity variable to 1 (food insecure) or 0 (food secure [ie, the counterfactual]) and the specific chronic condition variable to 1 (with chronic condition) while setting the value of all covariates at their mean. The same counterfactual approach was used to estimate the health care costs for those without the specific chronic condition by setting the specific chronic condition variable to 0. The mean differences between counterfactual conditions represent the incremental health care costs estimates. All estimations used the same model specification to quantify the incremental annual health care costs for each chronic condition (Appendix).

The dependent variable was total annual health care costs. The right side of the equation contained the food insecurity indicator and the chronic health condition indicator, their interaction, and a vector of statistical controls including age, sex, race/ethnicity, region, poverty level, health insurance status, adult living alone, SNAP participation, food-security modified version dummy, year, and a dummy for each other chronic condition. The interaction coefficient represents the difference-in-differences estimate, one of our coefficients of interest. Separate 2-part models were estimated for each of the chronic health conditions. We used bootstrapping with 1,000 repetitions to generate the 95% confidence intervals (CIs) (25,27). To reduce the influence of outliers, costs were capped at the 99th percentile of the costs distribution. We used STATA version 14.0 (STATA Corp) for all analyses.

## Results

We calculated the prevalence of chronic conditions and the characteristics of the analytic sample by food insecurity status (Table 1). Approximately 14% of older adults in our sample were food insecure (n = 2,150). Overall, prevalence of chronic conditions was higher for food-insecure older adults than for food-secure older adults, except for cancer, with all of these differences reaching significance (*P* ≤ .05). Three chronic conditions were most prevalent for both food-insecure and food-secure older adults: hypertension (59.7%, food insecure; 50.6% food secure), arthritis (49.5%, food insecure; 38.2% food secure), and diabetes (27.8% food insecure; 17.6%, food secure).

**Table 1:** Characteristics of Respondents (14,879) Aged 50 or Older by Food Security Status, 2013–2015 MEPS Linked With 2011–2014 NHIS^a^

Characteristic	Food Secure, % (n = 12,729)	Food Insecure, % (n = 2,150)	*P* Value^b^
**Age, y**			
50–64	51.5	68.1	<.001
65–85	48.5	31.9	
**Sex**			
Female	56.0	63.9	<.001
Male	43.0	36.1	
**Race/ethnicity**			
White, non-Hispanic	56.2	33.4	<.001
Black, non-Hispanic	19.6	34.3	
Hispanic	15.6	26.7	
Other, non-Hispanic	8.7	5.7	
**Living arrangement**			
Alone	40.0	51.0	<.001
Not Alone	60.0	49.0	
**Insurance**			
Private	60.2	27.3	<.001
Public	33.0	58.6	
None	6.8	14.2	
**Federal Poverty Level, %**			
<125	20.5	50.3	<.001
125–199	14.6	21.5	
≥200	64.9	28.2	
**Region**			
Northeast	18.0	15.4	<.001
Midwest	20.1	17.2	
South	37.8	45.4	
West	24.1	22.1	
**SNAP recipient**			
Recipient	11.5	44.4	<.001
Non-recipient	88.5	55.6	
**Chronic condition^c^**			
Hypertension	50.6	59.7	<.001
Coronary heart disease	8.3	11.9	
Stroke	5.1	9.8	
Emphysema	2.4	6.1	
Asthma	11.4	20.6	
Cancer	13.8	10.8	
Bronchitis	4.6	11.9	
Arthritis	38.2	49.5	
Diabetes	17.6	27.8	

Abbreviation: MEPS, Medical Expenditure Panel Survey; NHIS, National Health Interview Survey; SNAP, Supplemental Nutrition Assistance Program.

a All comparisons are based on unweighted data.

b *P *values are based on χ square test.

c Values do not total 100% because of comorbidity (ie, a respondent may have more than one chronic condition).

The distributions of sociodemographic characteristics between food-insecure and food-secure older adults were very different. Women were disproportionally overrepresented (63.9% for women vs 36.1% for men) among the food insecure, as were racial/ethnic minorities (non-Hispanic blacks and Hispanics). About 61% of food-insecure older adults were either non-Hispanic black or Hispanic. Half of food-insecure older adults were living below 125% of the Federal Poverty Level and about 45% lived in the South. Food-insecure older adults were also more likely to live alone and be uninsured or enrolled in Medicaid. All the sociodemographic differences between the groups reached significance (*P* ≤ .05).

We performed 2-part model estimations for each of the 9 chronic health conditions (Table 2) and calculated the average annual total health care costs for food-insecure and food-secure older adults with and without each chronic condition. For all 9 chronic health conditions, food-insecure older adults with a specific chronic condition incurred higher total health care costs than food-insecure adults without that specific condition and food secure adults with and without that specific chronic condition.

**Table 2:** Average Annual Total Health Care Costs Incurred by Food-Insecure and Food-Secure Adults Age 50 Years or Older, by Chronic Condition (US$ 2015)^a^^,^ b

Chronic Condition	With Chronic Condition		Without Chronic Condition	
	Food insecure	Food secure	Food insecure	Food secure
	Mean (95% Confidence Interval), $			
Hypertension	10,460 (9,070–12,160)	9,030 (8,640–9,460)	8,600 (7,610–9,900)	7,420 (6,980–7,960)
Coronary heart disease	12,380 (10,810–14,250)	11,480 (10,610–12540)	8,670 (7,930–9,620)	8,020 (7,720–8,340)
Stroke	13,290 (11,440–15,800)	11,900 (10,720–13,390)	9,080 (8,260–9,970)	8,120 (7,820–8,440)
Emphysema	10,600 (8,930–12,700)	9,710 (8,370–11,230)	9,160 (8,300–10,060)	8,380 (8,070–8,680)
Asthma	10,610 (9,280–12,100)	9,850 (9,120–10,680)	8,850 (8,040–9,790)	8,200 (7,870–8,530)
Cancer	10,240 (9,110–11,640)	9,710 (9,020–10,470)	8,710 (8,000–9,580)	8,250 (7,930–8,540)
Bronchitis	10,390 (8,970–12,150)	9,560 (8,580–10,720)	9,070 (8,230–9,830)	8,350 (8,090–8,680)
Arthritis	1,1240 (9,830–12,910)	9,500 (9,050–9,900)	8,710 (7,720–9,940)	7,370 (6,970–7,780)
Diabetes	12,430 (10,910–14,020)	11,180 (10,490–11,900)	8,470 (7,690–93,80)	7620 (7,280–7,950)

a Means adjusted for age, sex, race/ethnicity, insurance status, federal poverty level, geographic region, participation in Supplemental Nutrition Assistance Program, living alone, food security modified wording version, year fixed effects, and all other chronic conditions.

b Health care costs rounded to the nearest tenth.

Health care costs for food-insecure adults with a specific chronic condition ranged from $10,240 (cancer) to $13,290 (stroke); costs for food-secure older adults with the same chronic condition ranged from $9,030 (hypertension) to $11,900 (stroke). Health care costs for food-insecure adults without the specific chronic condition (counterfactual) ranged from $8,470 (diabetes) to $9,160 (emphysema) and for food-secure older adults without the specific chronic condition ranged from $7,370 (arthritis) to $8,380 (emphysema). Health care costs associated with food insecurity represented 18% of the incremental costs for adults with and without arthritis and 16% for adults with and without hypertension. On average, food insecurity added about 11 percent to the health care costs of older adults with and without a specific chronic condition.

We estimated the predicted food insecurity–related incremental annual total health care costs for each of the 9 chronic conditions (Table 3). We also calculated the difference-in-differences estimate (ie, the difference in health care costs associated with food insecurity between older adults with and without a specific chronic condition). Incremental costs associated with food insecurity among older adults with a specific chronic condition ranged from $530 (cancer) to $1,740 (arthritis); costs associated with hypertension, stroke, arthritis (*P* ≤ .05) and diabetes (*P* ≤ .10) reached significance. For those without the specific chronic condition, food insecurity–related incremental costs ranged from $460 for cancer to $1,340 for arthritis, showing significance for hypertension, stroke, arthritis (*P* ≤ .05), and diabetes (*P* ≤ .10).

**Table 3:** Total Average Annual Incremental Health Care Costs of Food Insecurity Among Adults Aged 50 or Older, by Chronic Condition, 2015^a^^,^ b

Chronic Condition	With Chronic Condition	Without Chronic Condition	Difference-in-Difference^d^
	Difference^c^	Difference^c^	
	Mean $ (95% Confidence Interval)		
Hypertension	1,420^e^ (20 to 3070)	1,190^e^ (30 to 2,570)	240 (–20 to 650)
Coronary heart disease	890 (–410 to 2,390)	650 (–280 to 1,680)	250 (–110 to 740)
Stroke	1,390^e^ (120 to 2,800)	950^e^ (80 to 1,910)	440^e^ (60 to 1,040)
Emphysema	900 (–110 to 2,100)	780 (–130 to 1,790)	120 (–20 to 510)
Asthma	760 (–390 to 2,010)	650 (–340 to 1,680)	120 (–70 to 400)
Cancer	530 (–350 to 1,780)	460 (–320 to 1,470)	70 (–50 to 330)
Bronchitis	820 (–210 to 1,880)	720 (–190 to 1,590)	100 (–20 to 410)
Arthritis	1,740^e^ (270 to 3,420)	1,340^e^ (230 to 2,580)	400^e^ (70 to 880)
Diabetes	1,250^f^ (–90 to 2,820)	850^f^ (–70 to 1,920)	390 (–20 to 940)

a Means adjusted for age, sex, race/ethnicity, insurance status, federal poverty level, geographic region, snap, living alone, food security modified wording version, year fixed effects, and all other chronic conditions.

b Health care costs rounded to the nearest tenth.

c The difference corresponds to the difference in health care costs between food-insecure and food-secure adults.

d The difference in health care costs associated with food insecurity between older adults with and without a specific chronic condition.

e Denotes statistical significance at *P* ≤ .05.

f Denotes statistical significance at *P* ≤ .10.

Difference-in-differences estimates showed food insecurity–related cost increments between adults with and without a chronic condition were as high as $440 for stroke, $400 for arthritis, and $390 for diabetes. These food insecurity–related additional costs were significant for stroke and arthritis (*P* ≤ .05) and were independent of the costs associated with experiencing food insecurity or having any chronic condition.

## Discussion

Our study showed that food-insecure older adults have higher costs than food-secure older adults with and without any of the selected chronic conditions. These findings highlight the financial burden of food insecurity in its own right. Even after controlling for the presence of other chronic conditions, food insecurity adds significant health care costs for older adults. We found positive and significant differences in the health care costs associated with food insecurity when comparing costs of older adults with and without hypertension, stroke, arthritis (*P* ≤ .05), and diabetes (*P* ≤ .10).

Our difference-in-differences estimates confirm that food insecurity interacts with chronic health conditions, generating higher health care costs among older adults. Our results suggest that food insecurity exacerbates the deleterious effects of chronic health conditions irrespective of the type of condition. Lack of adequate and reliable nutrition may be associated with people becoming more vulnerable to the advancement of illness, both biologically and at the level at which patients interact with the health care system. Food insecurity has been associated with delaying medical care, the timely and adequate intake of medications, and higher levels of service use such as emergency department visits and hospitalizations (28). Furthermore, food-insecure older adults appear to have more difficulty with the control and management of their chronic health conditions (20) and defer attending to their immediate health needs (29); both difficulties ultimately result in higher health care costs. Given the risks of going hungry, food-insecure adults also manifest a preference for unhealthy eating behaviors such as binge eating and consumption of high-calorie foods, which increase body fat, decrease muscle tissue, augment obesity and hypertension, and affect the endocrine, metabolic, catabolic, and cardiovascular systems (30). Our findings show that the interaction between food insecurity and chronic conditions is at the intersection of socioeconomic differentials in stress and biology. Food insecurity–related health care cost increments are high, both in absolute and relative terms.

Addressing food insecurity in this vulnerable population could achieve significant monetary health care savings and alleviate an unnecessary burden for older adults. Our results show that both food insecurity and chronic conditions are major drivers of health care costs. The alleviation of chronic conditions may not be immediate, but this is not the case for food insecurity. Food insecurity is socially determined; policies and programs targeting food insecurity could have a direct, substantial, and favorable effect on health outcomes and health care costs and use.

The importance of our findings are apparent when considering the size of the older adult (≥50 y) population in the United States and their increasing health care costs attributable to aging, food insecurity, and chronic conditions. Food–insecurity research shows that the health care costs associated with food insecurity in the United States were $160.7 billion in 2014 (12). About 36 million US adults aged 45 or older had one chronic health condition in 2012 (31). Another study found that in Washington, DC, alone the excess public health care cost for patients aged 65 or older with nutrition-related chronic conditions was $22.1 million for cancer and $96.5 million for diabetes 2010 (13). Although the demographics and populations in the Washington, DC, study vary from ours, our findings show a similar impact in terms of the high economic burden and health implications of food insecurity and chronic conditions.

Our study has several limitations. First, our analysis focuses on older adults who have one or more chronic conditions. Given that we control for the presence of each chronic condition and the interaction between food insecurity and a separate chronic condition, our study attempts to estimate and understand the economic burden of food insecurity as it interacts with a specific chronic condition, and our estimates are for each chronic condition separately. The economic burden of food insecurity as it relates to comorbidity is outside the scope of this study. Our results suggest, nevertheless, that such estimates are of great importance and should be addressed in future research, because a significant proportion of the adult population has more than one chronic condition. For instance, in 2012, approximately 52 million adults aged 45 or older had multiple chronic conditions (31). Second, in MEPS, costs associated with charitable care are not counted as expenditures. Considering that food-insecure older adults are more likely to receive charitable care than food-secure older adults, it is possible that some of the health care costs among food-insecure older adults were not captured in our estimates. Our estimates should therefore be viewed as conservative. Third, our models do not address causality or the direction of causality between food insecurity and health care costs; this is an important question that further research should address. Finally, NHIS is a random sample of participants interviewed throughout the year (of which MEPS includes a random sample); yet, food insecurity is assessed once and only for the last 30 days before the interview. Our estimated health care costs are annual, assuming that food insecurity is stable throughout cross sections of the data, and are representative of annual costs. This, however, may lead to biased health care costs estimates.

Policies and programs that address food insecurity in both the management and prevention of chronic health conditions have significant potential to reduce health care use and costs and improve the health and quality of life for older adults. The results of this study reveal only the direct health costs associated with food insecurity. There is still much that can be done to understand the full economic burden of food insecurity in older adults, how it interacts with chronic health conditions, and the value and impact of strategies to proactively address these issues in a rapidly evolving health care delivery system.

## Appendix

**Part I: Logistic regression model.** Equation 1 below outlines a logistic regression model, where *π_i_* denotes the probability of observing a positive health care cost value for the *i*-th observation with a specific chronic condition (ie, hypertension, coronary heart disease, stroke, emphysema, asthma, cancer, bronchitis, arthritis, or diabetes).

The econometric specification of part I of the model is of the following form:

logit(*π_i_*) = *β*_0 _+ *β*_1_
*x*_1_ + *β*_2_
*x*_2_ + *β*_3_
*x*_1_
*x*_2_ + *βz* + *ε_i_*

where *x*_1_ is a dummy for experiencing food insecurity, *x*_2 _is a dummy for a specific chronic health condition, *x*_1_ *x*_2 _represents the interaction between *x*_1_, and *x*_2, _and *z* is a vector of covariates, which includes age, sex, race/ethnicity, living arrangement, insurance, federal poverty level, region, SNAP recipient, food security modified version dummy, year fixed effects, and all other chronic conditions (1).

**Part II. Generalized linear model. **Equation 2 below outlines a generalized linear model with a log link, where *y_i_* denotes the health care costs for the *i*-th observation with a specific chronic condition (ie, hypertension, coronary heart disease, stroke, emphysema, asthma, cancer, bronchitis, arthritis, or diabetes) and that manifested a positive health care cost value.

The econometric specification of part II of the model is of the following form:

Log{E(*y_i_*)} = *β*_0_ + *β*_1_
*x*_1_ + *β*_2_
*x*_2_ + *β*_3_
*x*_1_
*x*_2_ + *βz* + *ε_i_,* x~F

where all variables in the right side of the equation represent the same set of variables implemented in Part I, and where F is the distributional family of *y_i_* (Poisson or gamma) (2).

For each chronic health condition, we used the product of the expectations from equations 1 and 2. From equation 1 we retrieved the probability of observing a positive health care cost *π_i_* = Pr(*y_i_* > 0|*x,* from equation 2 we retrieved the expected health care costs E(*y_i_*) = E(*y_i_|y_i_* > 0,*x*). The overall mean for each chronic condition can be written as follows (3):

E(*y_i_* |*x*) = Pr(*y_i_* > 0|*x*)
